# Effects of climate on the phenology of *Annona senegalensis* Pers. (Annonaceae) and the distribution of associated insects in Burkina Faso

**DOI:** 10.1002/ece3.70154

**Published:** 2024-08-09

**Authors:** Zézouma Anselme Dao, Rahim Romba, Bruno Jaloux, Amadé Ouedraogo, Olivier Gnankine

**Affiliations:** ^1^ Laboratoire d'Entomologie Fondamentale et Appliquée Université Joseph Ki‐Zerbo Ouagadougou Burkina Faso; ^2^ Institut Agro, Univ Rennes, INRAE, IGEPP Angers France; ^3^ Laboratoire de Biologie et Ecologie Végétales Université Joseph Ki‐Zerbo Ouagadougou Burkina Faso

**Keywords:** climatic variations, dry tropical zone, insect community, phenology, plant–insect interaction

## Abstract

Climate change and global warming in the Sahelian region cause dramatic drought and advancing of the desert. This phenomenon could affect the plant survival and community composition, but even for surviving plants, it could affect their phenology and the insect community associated with them. In a space‐for‐time approach, we studied the case of *Annona senegalensis* Pers. (Annonaceae), a common shrub in tropical areas, to determine the impact of climate change on its phenology and the insects associated with its flowers and fruits. We determined the phenology phases of *Annona senegalensis* during a 1‐year period and assessed the abundance and diversity of insects in the Sudanian and the Sudano‐Sahelian climatic zones of Burkina Faso. Temperature, rainfall and relative humidity were recorded during 12 months in two sites per zone. Leafing of *Annona senegalensis* lasted 10 months in the Sudanian zone, flowering and fruiting were 3 months long. In the Sudano‐Sahelian zone, leafing lasted 8 months while flowering and fruiting were 3 and 4 months long, respectively. A total of 10,040 insects belonging to 48 species were collected in the two climatic zones. Forty‐six species were found in the Sudanian zone while 25 species were recorded in the Sudano‐Sahelian one. The variations in the plant phenology and the insect community were mainly due to the variation in rainfall across both climatic zones. Our results emphasize that advancing of the desert due to climate change could not only affect the survival of plants but for resistant species it also affect their interactions with insects and the whole insect community associated.

## Graphical abstract

Climatic factors have an impact on the phenology of *Annona senegalensis* by shortening or lengthening the phenological phases in Burkina Faso. This affects the diversity and abundance of insects associated with this plant in cliamatic zones.

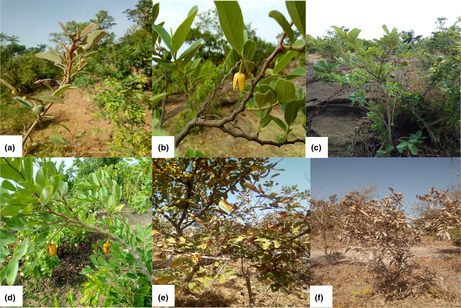

## 1 INTRODUCTION

The issue of climate change is one of the greatest concerns of our planet at the moment, in view of the numerous international meetings (IPCC, 2001, 2007). Global changes affect ecological processes, but the assessment of their impact on these processes remains difficult given the unprecedented speed of climate change currently recorded (IPCC, 2007). Ecological processes evolve over time, especially in a nonstationary environment (Damgaard, 2019). In the Sahelian zone, climate change is characterized by a reduction in precipitation and advancements of the desert that modifies the flora by excluding the species most sensitive to variations (Lompo et al., 2018; MECV, 2007). These modifications will also affect drought‐resistant plants, particularly their phenology by disrupting flowering and fruiting periods (MECV, 2007). These disruptions could, in turn, affect the community of flower‐visiting insects, pollinators, carpophagous insects, and their associated natural enemies, thus affecting the ecosystem services of pollination and plant pest regulation.

Climate variations affect in time and space the annual cycle of plants in tropical zones, which is characterized by alternating wet and dry seasons (Fournier, 1990). Due to the change in the annual cycle, woody plants are threatened in their natural environment by anthropic activities and pests. However, the role these plants play is essential in the agricultural systems of the dry zones of Africa because they can be considered as sources of fuel, food and an element of regulation of agro‐climatic conditions (Kouyaté, 2005). In addition, these plants are an important part of biodiversity by providing food resources and habitats for other species (MECV, 2007). Moreover, climate change represents a major potential threat to the viability of rural households that live mainly on the exploitation of natural resources in sub‐Saharan African countries, such as Burkina Faso (Brown & Crawford, 2008; Kaboré et al., 2019).

Reproductive phenology is crucial for the reproductive success and genetic diversity of plant species (Elzinga et al., 2007; Fox, 2003; Rathcke & Lacey, 1985). Phenology is also a crucial parameter in understanding the functioning of forest ecosystems, for measuring tree growth, and for monitoring plant adaptation to climate change (Bloesch & Viret, 2008). In Burkina Faso, *A. senegalensis* is a shrub species that plays an important role in food, nutrition and medicine for rural people (Arbonnier, 2009; Traoré et al., 2011; Zouré et al., 2021). Recent studies showed that the distribution of insects associated with *A. senegalensis* phenophases varies according to climatic zone (Dao et al., 2022). The causes of this variation are still unknown. We used a space‐for‐time substitution approach (Walker et al., 2010) to determine the impact of climate on *A. senegalensis* and on the insects associated with it in Burkina Faso. This approach is a method for studying slow ecological processes, where the relationship between ecological variables is studied in sites at different stages of development. It is widely used in biodiversity modeling to infer past or future trajectories of ecological systems from contemporary spatial patterns (Blois et al., 2013). This study method is therefore appropriate in a world shaken by climate variation to help further understand interregional climate variation, because it can help estimate and predict future changes.

In this study, we aimed to predict the future evolution of insect communities associated with *A. senegalensis* in the Sudanian region of Burkina Faso, based on the comparison of the climate conditions, plant phenology and insect diversity. We hypothesized that the geographical difference in *A. senegalensis* insect community could be linked to the influence of the local climate and plant phenology. We compared the past and current climatic parameters, the plant phenology, and the insect diversity and abundance in Sudanian and Sudano‐Sahelian climatic zones of Burkina Faso.

## 2 MATERIALS AND METHODS

### 2.1 Study area

**FIGURE 1:**
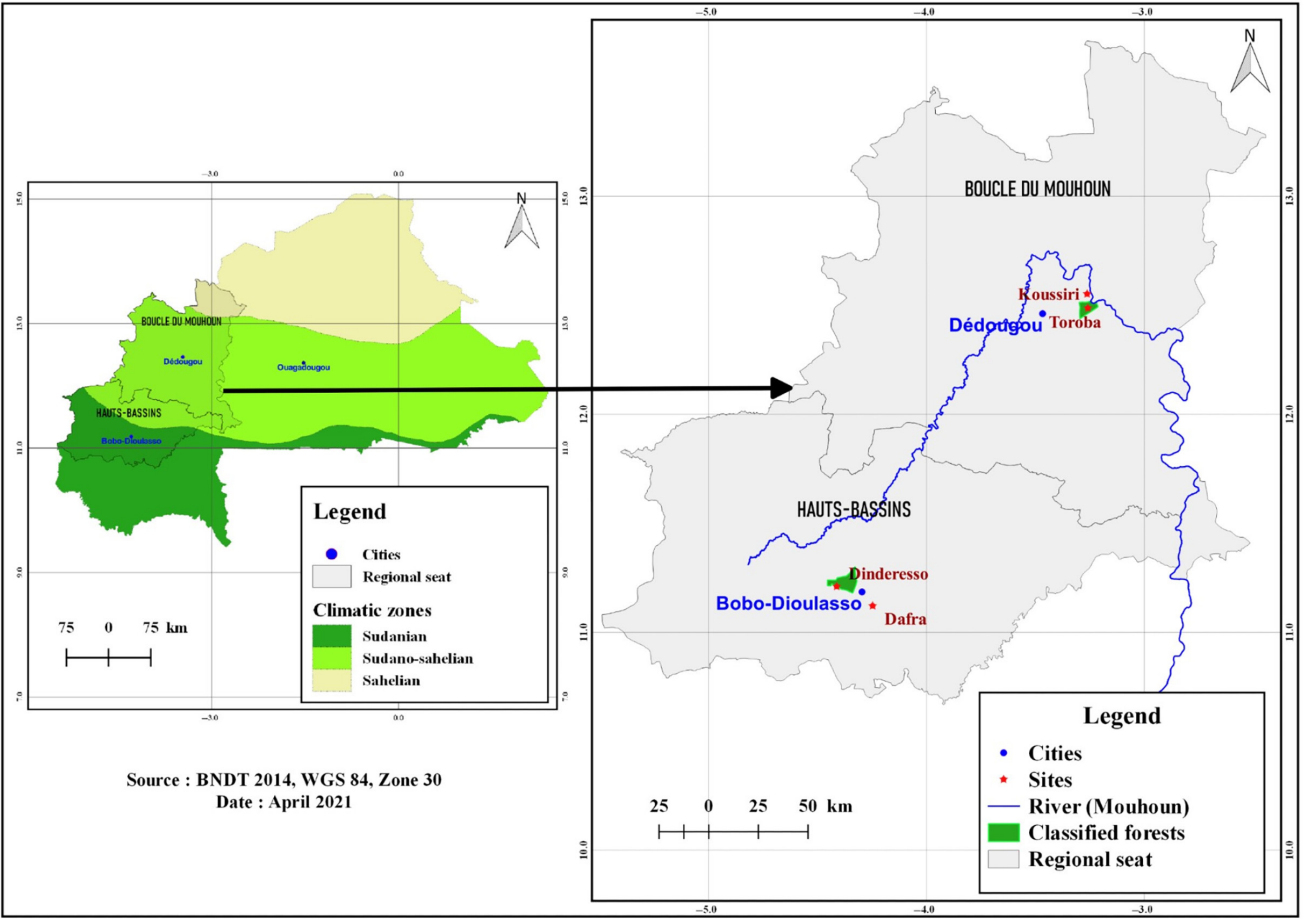
Map of the study area in Burkina Faso.

The study was conducted in Western Burkina Faso at the sites of Dindèrèsso classified forest (N 11°12′38″, W 004°24′43″) and Dafra wasteland (N 11°07′18″, W 004°14′50″) for the Sudanian climatic zone, and at the sites of Toroba classified forest (N 12°29′14″, W 003°15′35″) and Koussiri wasteland (N 12°33′09″, W 003°15′47″) for the Sudano‐Sahelian climatic zone (Figure 1), from May 2019 to May 2020. In each site, the average size studied was 1 km^2^.

Both study areas have dry tropical climate conditions, with two contrasting seasons: one dry season and one rainy season per year. Several biophysical characteristics differentiate the two climatic zones (Table 1).

**TABLE 1:** Biophysical characteristics of climatic zones.

Parameters	Sudano‐Sahelian	Sudanian
Annual rainfall (mm)	500–900	800–1100
Rainy months	From May/June to October	From May to October
Temperature range (°C)	22–32	25–30
Main vegetation types	Mosaic of savannahs	Dry forests, woodlands and savannahs

*Note*: Akoudjin et al. (2016) and Fontès and Guinko (1995).

### 2.2 Phenology assessment

To describe the phenology of the plants, the phenological phases of 60 shrubs of *A. senegalensis* were described per climatic zone, with 30 plants per site. During each phenology assessment, mature and nonmutilated *A. senegalensis* individuals were randomly sampled in the sites. The minimum distance between two plants was 100 m. Determination of the phenological phases of the plant was done by direct visual observation. During the active vegetation season, i.e., from May to October, observations were made in the morning every 10 days. In the dry season, the frequency of observations was 1 month, from November to April according to the method described by Grouzis and Sicot (1980) and Jaouadi et al. (2012).

Phenological characterization was carried out based on the benchmark methods described by Grouzis and Sicot (1980), Jaouadi et al. (2012) and Ouédraogo (1995). Leafing phase was defined as: L1: buds swelling, no developed leaves; L2: leaf buds + developed leaves (between 10% and 50% of the leaves on a plant); L3: mostly developed leaves (>50% of the leaves are green); L4: green leaves + dry leaves or leaves that have changed color in yellow (between 10% and 50% of the leaves); L5: >50% of the twigs on a plant have dry leaves, leaves falling.

Flowering phase: F1: flower buds only; F2: flower buds + opened flowers (between 10% and 50% of the flowers of a plant); F3: >50% of the branches have opened flowers; F4: opened flowers + dry flowers (>10% and <50%); F5: mostly dry flowers and falling of the flower parts.

Fruiting phase: G1: fruit set; G2: phase of growth of green fruits up to their maximum size (between 10% and 50% of the fruits); G3: >50% of the fruits are ripe; G4: ripe fruits + beginning of rotting; G5: fruits completely rotten and falling.

Stage 1 corresponds to the beginning and stage 5 to the end of each phase. The stages 2, 3, 4 represent the real phenology phase for an individual. Each of these three stages corresponds to the following intensities: beginning, optimum and end. Each phenophase and its different stages of development were characterized over the entire 12 months cycle. Other observations were made during the rainy season between May and October 2014, 2017, 2019, 2020, and 2021 in order to control leafing and flowering start times, the duration of flowering and fruiting in the studied areas.

### 2.3 Insect collection and identification

Samplings were done on the same sites (Dafra, Dindèrèsso, Koussiri, and Toroba) and at the same time as the phenology data collection. During each sampling, 30 mature plants per site and per phenological stage of *A. senegalensis* bearing flowers and/or fruits and growing in uncultivated areas were selected randomly. The insects were collected randomly on plants bearing flowers, green fruits, ripe fruits, and decayed fruits at a rate of 30 organs of 30 trees per stage and per site sampled according to the method described by Grouzis and Sicot (1980). The capture of insects was done either by immersion of the plant organ carrying crawling insects in 70% ethanol, by covering the plant organ using a plastic bag and beating in order to recover flying insects or by collecting insects encountered on the plant organ, according to the insect group present and their mobility (Dao et al., 2022). Insects from each organ were collected and stored in labeled bottles containing 70% ethanol or dried after collection (Lepidoptera).

Insects collected on different phenological stages of *A. senegalensis* were counted and examined in the laboratory under a binocular microscope using previously identified specimens by taxonomic entomologists or several taxonomic keys as Bland (1947), Cherix (1986), Chinery (1986), Pihan (1986), Stanek (1984), and Villiers (1943, 1977) and to perform identification of these insects. Some insects were identified by comparisons with reference collections housed at the Natural History Museum (London, United Kingdom) and the Swedish Museum of Natural History (Stockholm, Sweden).

### 2.4 Climatic data

The average temperature, relative humidity and rainfall recorded at the Bobo‐Dioulasso and Dédougou weather stations of the Agence Nationale de la Météorologie (ANAM) from 1975 to 1980 and from 2015 to 2020 were retrieved.

### 2.5 Data processing and analysis

#### 2.5.1 Phenology data analysis

The phenological spectrum was constructed by calculating, for each observation date, the frequencies within the population of individuals in the leafing (L%), flowering (F%) and fruiting (G%) phases (Grouzis & Sicot, 1980; Jaouadi et al., 2012). *P* (%) = ni/*N* × 100 is the relationship, with *P* (%): percentage of individuals at the site present at the various phases of leafing, flowering or fruiting; ni: number of individuals presenting a given phenological stage; *N*: total number of sampled individuals (population size, i.e. 30 plants). The frequency of phenological stages was calculated for each observation. Studied parameters were the percentages of trees in flowering (null: 0%; weak: 1–20%; average: 20–60%; intense >60%), leafing (average < 50%; intense >50%) and fruiting (null: 0%; weak: 1–20%; average: 20–60%; intense >60%).

### 2.6 Statistical analysis

Statistical analyses were performed on sites and phenological stages with the R software version 4.0.3. Shannon's diversity (specific richness) and Pielou equity (regularity of the distribution of individuals between species) indices were calculated for insect community (Shannon & Weaver, 1949). The formulas used to calculate these indices are as follows: $H=-\sum_{i=1}^{S}\mathrm{pi}\;\log_{2}\mathrm{pi}$ (Shannon diversity index) and $E_{H}=\frac{H}{H\max}$ (Pielou equity index); EH ranges between 0 (a single species has a probability of 1) and 1 (all species have the same probability), where *S* = total number of species per zone or per stage; pi = (ni/*N*), relative frequency of species; ni = relative frequency of species i in a collection site or for a zone; *N* = sum of specific relative frequencies; *H*max = ln S.

The Kruskal–Wallis test was used to compare the average abundance and specific diversity of insects by climatic zone and the χ^2^ test for the distribution of flower insects. Statistical analyses were first carried out on the proportions of plants in each phenological stage according to the date and site in the climatic zones studied. In order to test for an effect of the climatic zone on the durations of the phenological phases, a Fisher's exact test was used to compare the proportions of plants in each leafing, flowering and fruiting stage in the two climatic zones. We also used the Mann–Whitney test both to compare mean temperatures, relative humidity, and rainfall between climatic zones and between years (1975–1980 and 2015–2020). For all these tests, a 5% probability threshold was chosen.

In addition, the Pearson correlation test *r* (*r* is the correlation coefficient, between −1 and 1, with a strong correlation when r tends toward 1 or −1, and a weak correlation when r tends towards 0) was also calculated between each climatic parameter and each phenophase of each climatic zone to find out whether they are related or independent.

## 3 RESULTS

### 3.1 Climate parameters

#### 3.1.1 Temperature

**FIGURE 2:**
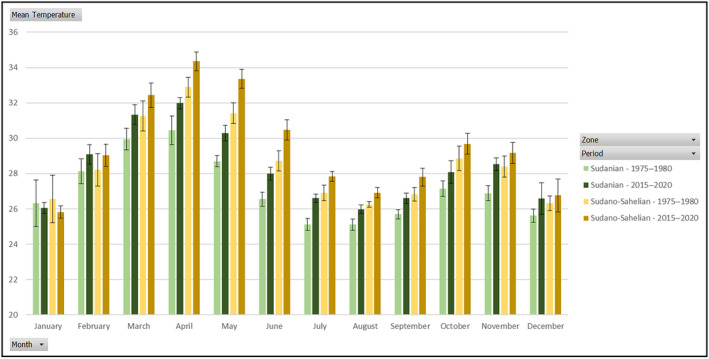
Variation of monthly mean temperatures from 1975 to 1980 and from 2015 to 2020 in the Sudanian and Sudano‐Sahelian climate zones.

Data from 1975 to 1980 and from 2015 to 2020 showed that mean temperature ranges were high in both climatic zones (26–34°C) in western Burkina Faso. However, they were slightly cooler in the Sudanian zone. The highest temperatures were recorded between the months of March and May, during the dry season. The lowest temperatures were observed between July and September in the rainy season and also between December and January in the dry season (Figure 2). There is a difference between the two periods for each zone, but 1975–1980 mean temperatures in the Sudano‐Sahelian zone were similar to those of 2015–2020 in the Sudanian zone (Mann–Whitney, *p* = .41).

#### 3.1.2 Relative humidity

**FIGURE 3:**
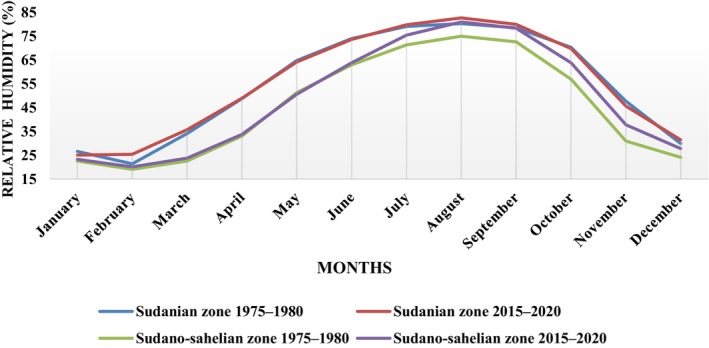
Variation of average relative humidity from 1975 to 1980 and from 2015 to 2020 in the Sudanian and Sudano‐Sahelian climatic zones.

The highest relative humidity rates were recorded between July and September corresponding to the full rainy season and the lowest between December and March during the dry season. Relative humidity did not vary significantly over the past 40 years in either climate zones (Mann–Whitney, *p* = .34), although it was sometimes slightly higher in the Sudanian zone (Figure 3).

#### 3.1.3 Rainfall

**FIGURE 4:**
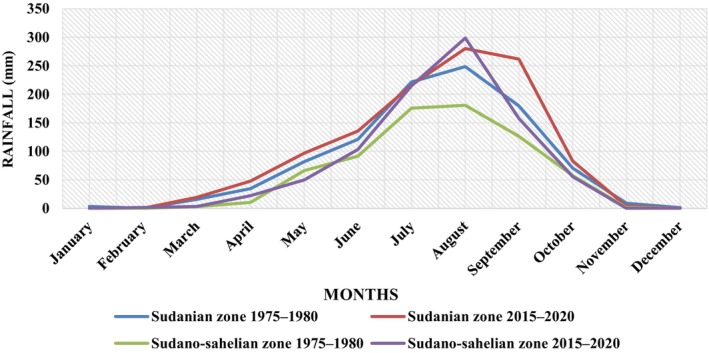
Compared variation of average rainfall from 1975 to 1980 and from 2015 to 2020 in the Sudanian and Sudano‐Sahelian climatic zones.

According to our data, a comparison between rainfall in the Sudanian and the Sudano‐Sahelian climate zones in Burkina Faso showed that rainfall did not vary (Mann–Whitney, *p* = .25 in all cases). But visibly, rainfall tended to be higher in 2020 compared to 1980 (Figure 4). The wettest months were from July, August, and September. But it did not usually rain between November and February. The lowest rainfall was recorded during the years 1975–1980 in the Sudano‐Sahelian climate zone.

### 3.2 Phenology of *A. senegalensis* across the two climatic zones of Burkina Faso

#### 3.2.1 Phenological calendar of *A. senegalensis*

**FIGURE 5:**
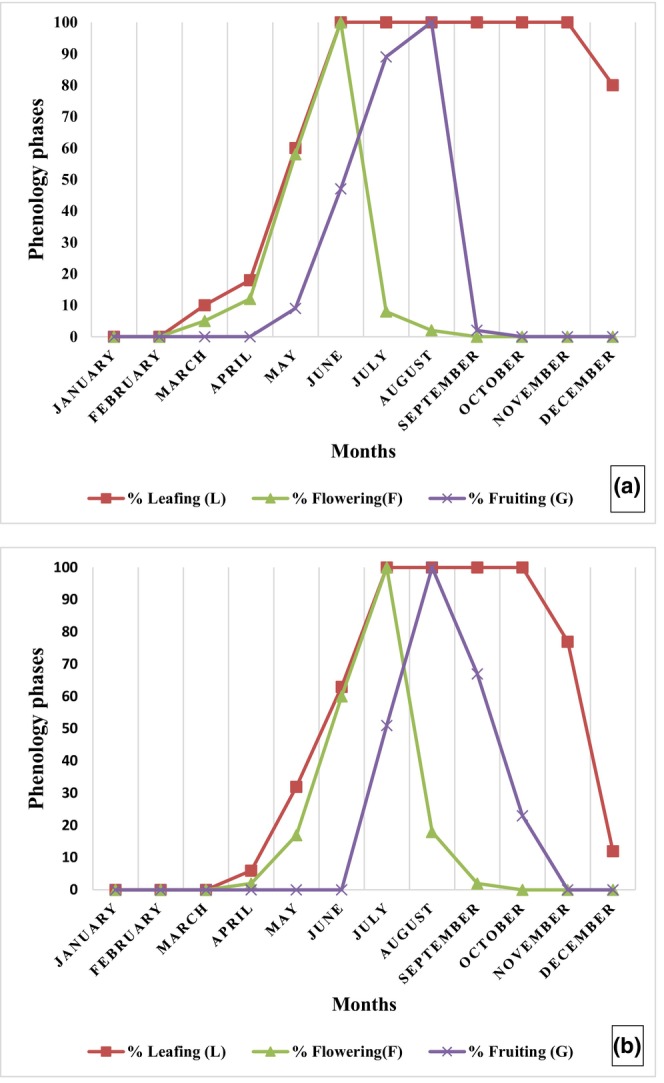
Phenophases of *Annona senegalensis* in the Sudanian (a) and Sudano‐Sahelian climatic zones (b) from 2019 to 2020.

In the Sudanian zone, the plant bore leaves during 10 months per year, from April to January (Figure 5a), whereas in the Sudano‐Sahelian zone, the leafing period lasted 8 months, from May to December (Figure 5b). The optimum leafing period when the plant had full leaves occurred between June and November (5 months) in the Sudanian zone, and from July to October in the Sudano‐Sahelian zone (4 months).

Flowering lasted 4 months, from April to July, and the optimum flowering stage was observed between May and June in the Sudanian zone. Flowering was spread over May to August (4 months) and reached its optimum stage between June and July in the Sudano‐Sahelian zone.

*Annona senegalensis* bore fruit from the end of May to the end of August (3 months), with optimum fruiting from June to July in the Sudanian zone. Fruiting covered July to October (4 months), with the optimum between August and September in the Sudano‐Sahelian zone.

**FIGURE 6:**
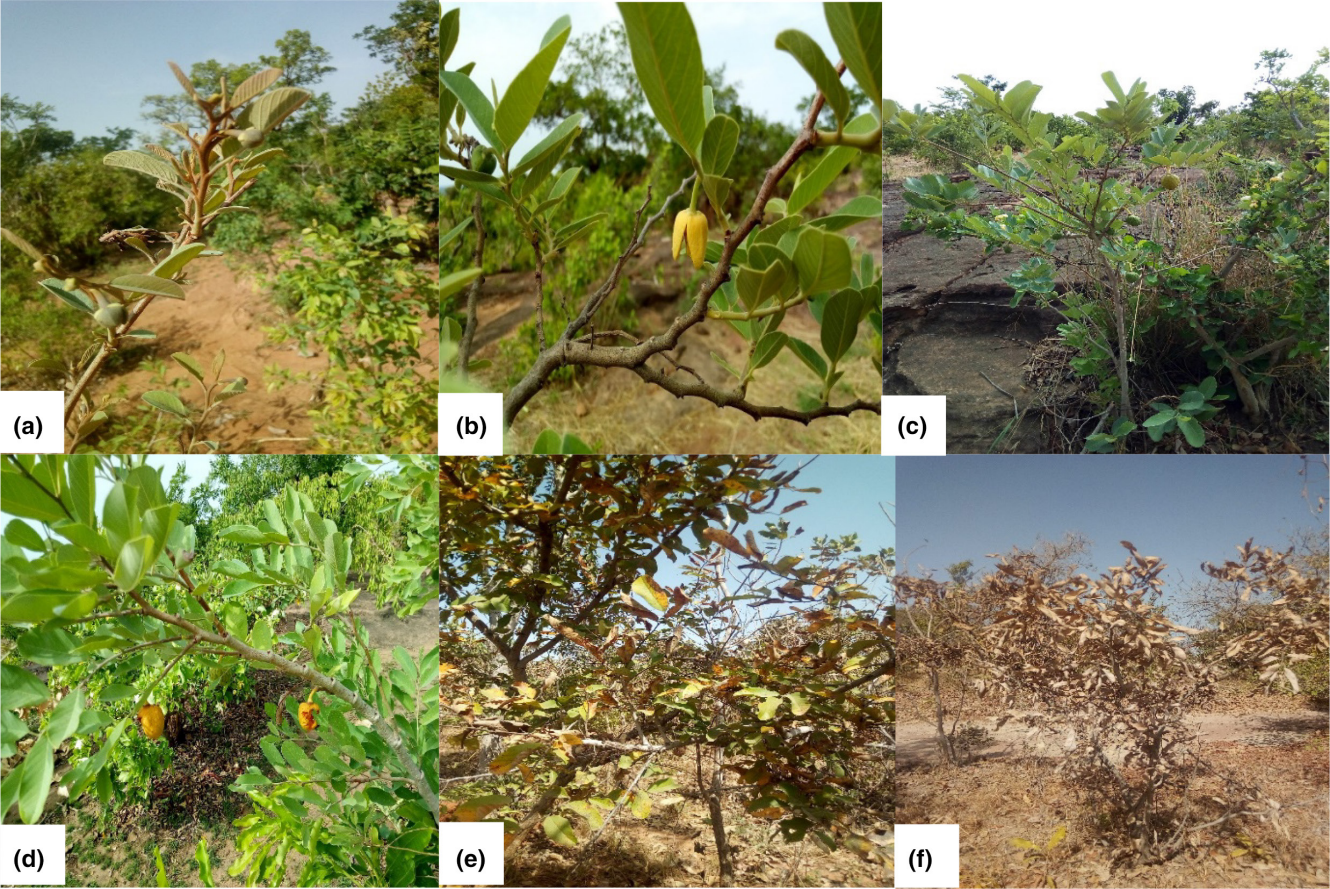
Some phenological stages of *Annona senegalensis* in western Burkina Faso. (a) L1 (buds swelling) and F1 (flower buds only) stages; (b) L2–L3 (developed leaves and mostly developed leaves) and F2–F3 (flower buds + opened flowers) stages; (c) L3 (mostly developed leaves) and G2 (phase of evolution of green fruits) stages; (d) L3 and G3 (ripe fruits) stages; (e) L4 (green and dry leaves) stage; (f) L5 (dry leaves and leaves falling) stage.

The phenological stages of *A. senegalensis* did not occur at the same time during the year in the Sudanian and the Sudano‐Sahelian climatic zones (Figure 6). The leaves and flowers of *A. senegalensis* appeared simultaneously in each climatic zone. Leafing and flowering duration were significantly different (Fisher, respectively *p* < .05) in the two climatic zones. But no significant difference was observed between the fruiting duration in both climatic zones (Fisher, *p* = .15).

There was a shift in the onset of phenology phases between the Sudanian and the Sudano‐Sahelian zones. Leafing began in April in the Sudanian zone while it began in May in the Sudano‐Sahelian zone. Flowering began in April in the Sudanian zone, whereas it began in May in the Sudano‐Sahelian zone. Fruiting of *A. senegalensis* started in May in the Sudanian zone, whereas it began in June in the Sudano‐Sahelian zone, i.e., 2 months later.

#### 3.2.2 Interactions between climatic factors and phenophases

In the Sudanian zone, fruiting was correlated with temperature and relative humidity: an increase in temperature implied a decrease in the duration of fruiting (*r* < −0.7) and the duration of fruiting increased with increasing relative humidity (*r* > 0.7) and rainfall (*r* > 0.73).

In the Sudano‐Sahelian zone, the flowering duration was strongly related to temperature, with the duration increasing with increasing temperature (*r* > 0.81). The leafing and fruiting durations also increased with rainfall (respectively *r* > 0.74 and *r* > 0.92). Likewise, the duration of leafing increased when relative humidity increased (*r* > 0.91).

Observation of the phenology of *A. senegalensis* showed that leaves and flowers appeared only when it rained in the area concerned, whereas flowering and fruiting could be shortened or lengthened, respectively, if it rained regularly or if rainfall was scarce.

### 3.3 Climate impact on the distribution of insects associated with *A. senegalensis*

#### 3.3.1 Insect diversity in the climatic zones

In the Sudanian and Sudano‐Sahelian climatic zones, 48 insect species from 23 families and 6 orders were collected and identified on the flowers and fruits of *A. senegalensis*. 24 insect species were specifically found in the Sudanian zone and two others were specifically found in the Sudano‐Sahelian zone (Table 2).

**TABLE 2:** Abundance of insect species of *Annona senegalensis* in the two climatic zones.

Order	Family	Species	Species number in Sudanian zone	Species number in Sudano‐Sahelian zone
Orthoptera	Acrididae	*Catantops stylifer* Krauss	3	0
Hemiptera	Coreidae	*Clavigralla* sp.	1	0
	Pentatomidae	*Staria lunata* Hahn	19	44
	Pyrrhocoridae	*Dysdercus voelkeri* Schmidt	6	17
	Tettigometridae	*Hilda undata* Walker	653	58
Hymenoptera	Apidae	*Apis mellifera* Linnaeus	12	0
	Figitidae	*Leptopilina* sp.	2	0
	Formicidae	*Brachyponera sennaarensis* Mayr	143	9
		*Camponotus maculatus* Fabricius	29	0
		*Camponotus sericeus* Fabricius	10	2
		*Cataglyphis bombycina* Roger	1	0
		*Crematogaster* sp.	366	638
		*Messor galla* Mayr	693	4
		*Monomorium* sp.	11	3
		*Oecophylla longinoda* Latreille	7	0
		*Pogonomyrmex barbatus* Smith	102	30
		*Trichomyrmex abyssinicus* Forel	87	1307
	Vespidae	*Polistes bellicosus* Cresson	1	0
		*Ropalidia capensis* Saussure	1	0
	Chrysomelidae	*Aulacophora* sp.	2	5
	Curculionidae	*Endaeus* spp.	1574	378
	Nitidulidae	*Carpophilus nepos* Murray	846	608
		*Soronia punctatissima* Illiger	8	0
	Scarabaeidae	*Charadronota quadrisignata* Gory & Percheron	3	0
		*Diplognatha gagates* Forster	9	1
		*Leucocelis nitidula* Olivier	1	0
Coleoptera	Scarabaeidae	*Leucocelis puncticolis* Moser	15	2
		*Myodermum alutaceum* Afzelius	3	9
		*Pachnoda ardoini* Ruter	0	4
		*Pachnoda cordata* Drury	0	2
		*Pachnoda* sp.	4	0
		*Polybaphes aequinoctialis* Olivier	5	1
		*Polybaphes sanguinolenta* Olivier	38	12
		*Rhabdotis sobrina* Gory & Percheron	8	0
		*Uloptera planata* Burmeister	1	0
		*Xeloma maura* Boheman	17	22
	Staphylinidae	*Philonthus marginatus* Müller	9	0
Lepidoptera	Lycaenidae	*Myrina silenus* Fabricius	1	0
	Nymphalidae	*Charaxes epijasius* Reiche	1	0
Diptera	Bibionidae	*Dilophus febrilis* Linnaeus	1	0
	Calliphoridae	*Calliphora vomitoria* Linnaeus	9	0
		*Pollenia rudis* Fabricius	2	0
	Chloropidae	*Hippelates pusio* Loew	7	0
	Drosophilidae	*Zaprionus indianus* Gupta	1646	488
	Muscidae	*Musca domestica* Linnaeus	2	0
	Tephritidae	*Bactrocera dorsalis* Hendel	2	0
		*Ceratitis cosyra* Walker	17	11
	Ulidiidae	*Physiphora alceae* Preyssler	5	1

#### 3.3.2 Distribution of insects in the climatic zones

A total of 10,040 insects belonging to the 48 insect species were collected at the four study sites and distributed across the two climatic zones. Insects were significantly more diverse and more abundant in the Sudanian zone than the Sudano‐Sahelian one (Kruskall‐Wallis, *p* < .05; Table 3). The diversity indexes associated were high whatever in the Sudanian zone and slightly lower in the Sudano‐Sahelian zone, showing the richness of the insect community associated with *A. senegalensis*.

**TABLE 3:** Distribution of insects associated with *Annona senegalensis* in the climatic zones.

	Richness	Abundance	Shannon diversity index	Pielou equity index	Richness	Abundance
Sudanian zone	46	6383	4.23	0.76	Kruskall‐Wallis; *p* < .05	Kruskall‐Wallis; *p* < .05
Sudano‐Sahelian zone	25	3657	3.32	0.71		

#### 3.3.3 Distribution of insects in the sites

In each climatic zone, insects were diverse and equally distributed. Moreover, they were more abundant in the classified forest sites than wasteland sites (Table 4).

**TABLE 4:** Distribution of insects associated with *Annona senegalensis* according to site type.

	Climatic zone	Sites	Abundance	Shannon diversity	Pielou equity
Classified forests	Sudanian	Dindèrèsso	3485	3.97	0.75
	Sudano‐Sahelian	Toroba	2160	3.41	0.75
Wastelands	Sudanian	Dafra	2897	4.30	0.80
	Sudano‐sahelian	Koussiri	1497	2.89	0.70

**FIGURE 7:**
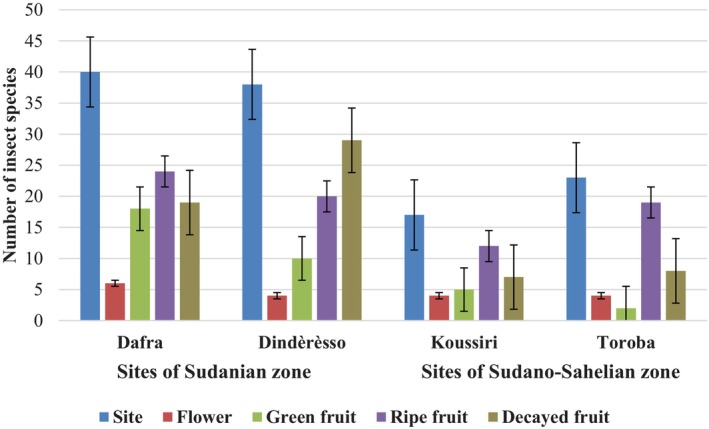
Distribution of insect species number in connection with site and phenological stages.

The difference in species richness was even more important in the insect community associated with different fruit types (ripe, green and decayed) where the insect species richness was much higher than on the flowers (χ^2^, *p* < .05; Figure 7). The size of this community was smaller in the Sudano‐Sahelian zone (Koussiri and Toroba), where the leafing duration was shorter and the fruiting duration was longer than in the Sudanian zone (Dafra et Dindèrèsso). However, there was no significant variation in the proportion of insect communities associated with flowering in both climatic zones (χ^2^, *p* = .56).

**FIGURE 8:**
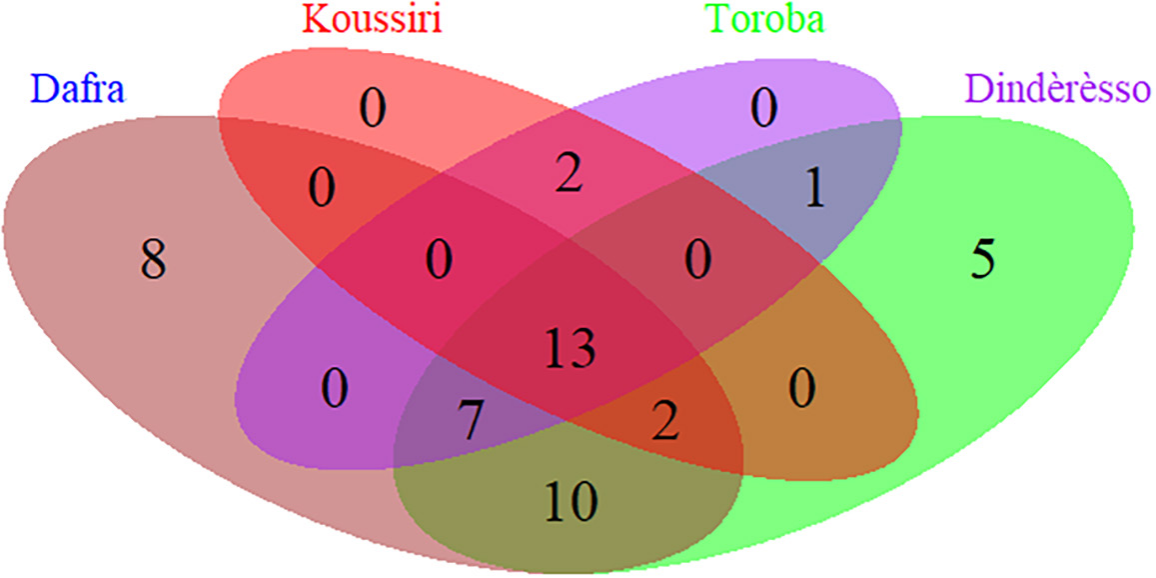
Insect species similarity per site in the Sudanian and the Sudano‐Sahelian climatic zones.

Thirteen insect species are common to all four sites. However, some insects are unique to a given site. For example, the Dafra site (Sudanian zone) contained eight unique species not found in any of the other sites. The same applies to the Dindèrèsso site (Sudanian zone) with five unique species and the Toroba (Sudano‐Sahelian) site, with one unique species (Figure 8).

## 4 DISCUSSION

During the period 1975–1980, temperatures in the Sudanian zone were similar to those of the 2015–2020 period in the Sudano‐Sahelian zone of Burkina Faso. This observation provides evidence that temperatures vary over time and space. In view of the temperature rise in both climatic zones over the last 40 years, temperatures in the Sudanian climatic zone are likely to be similar to the current ones in the Sudano‐Sahelian zone. And so, the high temperatures in the Sudano‐Sahelian zone could directly affect insect survival, exposing them more frequently to lethal heat, on a daily or several days scale, and water depletion. Exposure to extreme temperatures can have negative impacts on insect diversity and abundance (Ma et al., 2021), leading in some cases to local extinctions (Harvey et al., 2023; Thakur et al., 2022). Indeed, insect development and survivorship increase with increasing temperatures, but with species‐specific upper thermal optima, lethal and sublethal effects increase (Hance et al., 2007; Prado et al., 2010; Scriber & Slansky, 1981). Developmental rate begins to decrease at supraoptimal temperatures (Sharpe & DeMichele, 1977; Thakur et al., 2022). This could lead them to migrate to relative cooler environments like in the Sudanian zone, which would be more favorable to their living conditions, or to simply disappear from the Sudano‐Sahelian zone. Since insects are very sensitive to temperature variations, each species has its own requirements with certain temperature ranges that are favorable for their survival and development (Nageleisen, 2018). And then, temperature variations over the year determine plant phenology over time, and indirectly condition the resources available to insects. Supraoptimal temperature in plant could lead to leaf drying, premature falling, sap flow stop and alter the food resource for the insects (Rehman et al., 2019), contributing to a decline in insect populations (Soro et al., 2020).

Several insect species would have more favorable living conditions such as more suitable rainfall in the Sudanian zone, along with the availability of food resources such as leaves, flowers and fruit, which occur earlier in the Sudanian zone. The rainfall and relative humidity are slightly higher in the Sudanian zone. However, an increase in relative humidity has a negative effect on insect populations (Soro et al., 2020) and a positive effect on fruiting. If rainfall decreases by 7.3% in 2050 in the region, as predicted by MECV (2007), the biomass potential would decrease considerably, which can lead to the disappearance of certain species (plant and animal) and the migration of other species from the Sahelian to the Sudanian regions of Burkina Faso. Therefore, a significant loss of the existing insect community in the Sudanian zone due to climatic variations could make it resembling the current Sudano‐Sahelian zone in the future. So, a large number of insect species would have disappeared or migrated to areas more favorable to their life. Moreover, the study of the *A. senegalensis* phenology showed that its life cycle is dependent on rainfall, and the climatic zone significantly influences its phenology. We note a loss of leaves in the dry season, a stop of fruiting before the end of the rainy season, and the onset of leafing and flowering at the beginning of the rainy season. Flowering and fruiting reach their optimum stage in June or July when the rains fall sufficiently, but their durations are extended when the rains become scarce. Indeed, the phenological cycles of tropical trees are influenced by climatic factors (Breman & Kessler, 1995; Chuine et al., 2000; Peres, 1994; Poupon, 1979; Sun et al., 1996).

There is a higher species diversity and abundance of insects in the Sudanian zone than in the Sudano‐Sahelian one (Dao et al., 2022, 2023). In addition, insect populations in protected areas are more diverse and more abundant on fruits and flowers compared to those on wasteland sites. The size of natural habitats and their botanical quality are two factors that strongly influence plant and entomological diversity (Bessat et al., 2019). Then, protected areas are far from phytosanitary treatment fields unlike wasteland sites, where insect populations would be affected by pesticides used in the vicinity. Tropical forests degradation is one of the principal factors of insect decline (Wagner et al., 2021). Otherwise, the phenology of the same species can vary significantly from one environment to another one, and the adaptation of the community of woody species to the conditions of the environment is usually done by a floristic modification (Fournier, 1990). Thus, leafing and flowering begin earlier in the Sudanian zone where *A. senegalensis* has a longer leafing phase than in the Sudano‐Sahelian zone, while fruiting is longer in the Sudano‐Sahelian zone compared to the Sudanian zone. Studies aimed at assessing species occupancy patterns must consider their spatial variation when drawing conclusions about the relationship between species and their biotope (Srivathsa et al., 2018).

*Annona senegalensis* bears leaves for a long time of the year (at least 8 months) in both climatic zones. Leaves being the basis of the plant's metabolism, evergreenness is therefore a fundamental attribute in the functioning of the plant (Segrestin, 1995). However, most savanna species generally lose their leaves in the dry season (Clanet & Gillet, 1980; Sanogo, 1997). Our results corroborate those of Rasamimanana et al. (2012) who showed that leaves appear at the beginning of the rainy season. The simultaneous appearance of leaves and flowers in *A. senegalensis* is typical of woody plants (Fournier, 1990). The coevolution between trees and the associated insects, called phenological coincidence, is a synchronization between the food resource availability (such as leaves) and the occurrence of the life stages of the insects (young caterpillars or larvae) that will feed on these newly formed organs (Nageleisen, 2018). The shift in phenological phases of the *Annona senegalensis* may result in a disruption of this synchronization, at a rate too high and too fast to allow adaptation of interacting insects species.

## 5 CONCLUSION

The phenological calendar of *Annona senegalensis* in Burkina Faso showed that leafing and fruiting are longer in the Sudanian climatic zone, whereas fruiting is longer in the Sudano‐Sahelian zone, with a difference in the beginning of the phenological phases. The phenophases were correlated with climatic factors, in particular temperature and rainfall. Climatic conditions were more favorable to insect diversity and abundance in the Sudanian zone than the Sudano‐Sahelian one. These variations in insect distribution and phenology of *A. senegalensis* in the two climatic zones are mainly due to the distribution of rainfall in time and space.

Climate change will have an impact on the phenology of *A. senegalensis* by shortening or lengthening the phenological phases, which will probably affect the diversity and abundance of insects associated with these plants. Biodiversity conservation strategies should consider this phenomenon to mitigate this.

## AUTHOR CONTRIBUTIONS

**Zézouma Anselme Dao:** Conceptualization (equal); data curation (equal); formal analysis (equal); investigation (equal); methodology (equal); project administration (equal); resources (equal); software (equal); visualization (equal); writing – original draft (equal); writing – review and editing (equal). **Rahim Romba:** Formal analysis (equal); methodology (equal); supervision (equal); validation (equal); writing – review and editing (equal). **Bruno Jaloux:** Formal analysis (equal); methodology (equal); project administration (equal); validation (equal); writing – review and editing (equal). **Amadé Ouedraogo:** Conceptualization (equal); investigation (equal); methodology (equal); project administration (equal); supervision (equal); validation (equal); visualization (equal); writing – review and editing (equal). **Olivier Gnankine:** Conceptualization (equal); project administration (equal); resources (equal); supervision (equal); validation (equal); visualization (equal); writing – review and editing (equal).

## CONFLICT OF INTEREST STATEMENT

The authors have no relevant financial or nonfinancial interests to disclose.

## Data Availability

The datasets analyzed during the current study were provided as supplementary files.
